# Lesson learned from implementing measures to prevent urinary tract infection and bladder distension in patients with hip fractures - a process evaluation

**DOI:** 10.1186/s12877-025-06216-w

**Published:** 2025-08-02

**Authors:** Maria Frödin, Brigid M. Gillespie, Ewa Wikström, Cecilia Rogmark, Bengt Nellgård, Annette Erichsen

**Affiliations:** 1 Institute of Health and Care Sciences, Sahlgrenska Academy, University of Gothenburg, Sahlgrenska University Hospital/Mölndal, Operation 1, Göteborgsvägen 31, Göteborg, SE-431 80 Sweden; 2 NMHRC Centre of Research Excellence in Wiser Wound Care, School of Nursing & Midwifery, Griffith University, Griffith, QLD Australia; 3 Gold Coast University Hospital and Health Service, Gold Coast, Australia; 4 Department of Business Administration, School of Business, Economics and Law, University of Gothenburg, Gothenburg, Sweden; 5 Department of Orthopedics Malmö, Skane University Hospital, Lund University, Lund, Sweden; 6 Swedish Arthroplasty Register, Centre of Registers Västra Götaland, Gothenburg, Sweden; 7 Department of Anesthesiology and Intensive Care Medicine, Institute of Clinical Sciences, Sahlgrenska University Hospital, University of Gothenburg, Gothenburg, Sweden; 8 Department of Orthopedics, Sahlgrenska University Hospital, Gothenburg, Sweden

**Keywords:** Process evaluation, Integrated knowledge translation, Urinary catheter-associated urinary tract infections, Hip fracture, Bladder distension, Implementation intervention

## Abstract

**Background:**

Catheter-associated urinary tract infections and bladder distension are common and preventable adverse events. This study presents a process evaluation of a bladder bundle, designed to change healthcare professionals’ way of thinking and acting to prevent these adverse events, using theories of organizational culture, leadership, and an integrated knowledge translation approach.

**Aim:**

To enhance understanding of barriers and enablers when implementing recommendations to prevent catheter-associated urinary tract infections and bladder distension. We examined the implementation concepts of feasibility, acceptability and fidelity, guided by the following research questions: (1) To what extent was the intervention delivered as planned? (2) What factors influenced the implementation process, and how did these factors influence implementation outcomes?

**Methods:**

A qualitative and quantitative process evaluation was conducted, guided by the Medical Research Council framework. The intervention was implemented between 2016 and 2020, in a Swedish university hospital, across six units involved in hip fracture care. Data was collected through field notes, implementation logs, emails, presentations, and attendance records. Qualitative data were analyzed using deductive and inductive content analysis. Quantitative data, including attendance and adherence rates, were descriptively summarized under the categories of fidelity, dose, reach, context, and mechanisms of impact.

**Findings:**

The implementation of the intervention was successful regarding feasibility, acceptability and fidelity, which is important for adoption and ownership of interventions. Factors that triggered change were feedback on patient outcomes and ensuring time for learning and re-learning in a safe milieu. Barriers to the intervention were shortages in the workforce, production pressures and lack of experience in collaboration in change projects involving different organizational units. The implementation program enabled ways to work around barriers on macro, meso and micro levels in the organization.

**Conclusion:**

Implementing practices to prevent both UC-UTIs and bladder distension is feasible but complex and time-intensive. Using theories of organizational culture and leadership, along with a collaborative approach, can support adoption and sustainability of best practices in complex healthcare settings. Findings offer insights for healthcare decision-makers aiming to improve catheter care.

**Trail registration:**

Clinical Trial Registry ISRCTN 17,022,695, retrospectively registered on 23 December 2021, after data collection was completed.

**Supplementary Information:**

The online version contains supplementary material available at 10.1186/s12877-025-06216-w.

## Background

Urinary catheter-associated urinary tract infections (UC-UTIs) and bladder distension are largely preventable adverse events if healthcare professionals (HCPs) adhere to recommended best practices [1, 2]. Research suggests that up to 50–70% of UC-UTIs [3, 4], and as many as 90% of bladder distension cases are preventable [4]. These adverse events are not only highly interrelated [1, 5],, they are also common in older patients with hip fracture [6, 7]. The high prevalence of patients with bladder distension in orthopedic patients may stem from an overly cautious approach to indwelling urinary catheters (IUCs) use [4]. This highlights the need for integrated preventive strategies that address both complications [4]. However, existing UC-UTI prevention bundles often fail to consider the risk of bladder overdistension associated with overly restrictive IUC use [1, 2].

Despite the availability of evidence-based recommendations, consistent implementation in clinical practice remains a challenge. The adoption and routinization of best practices in hospitals often vary, reflecting a persistent implementation gap [8, 9] and implementation are frequently unsuccessful [10]. One major challenge is the time lag between the initial error and the appearance of symptoms, which limits timely feedback and makes it harder for HCPs to connect cause and effect, thus reducing motivation to change [11].

Process evaluation can shed light on how and why complex intervention succeed or fail, by exploring their mechanism of impact and contextual factors influencing outcome(s) [8, 9]. This knowledge is valuable to both decision-makers, who can use it to guide implementation strategies, and researchers seeking to replicate or refine interventions. Yet, there are few interventions that aim to prevent UC-UTIs present process evaluation findings [2, 3, 12–14].

Over a five-year period (2015–2020) we conducted two interrelated, theory-driven implementation interventions, *Safe Hands* [15, 16] and *Safe Bladder* [6, 7]. Designed to change HCPs way of thinking and acting to prevent UC-UTIs and bladder distension in patients with hip fracture. The interventions were informed by theories on organizational culture, and dialogue [17, 18] focusing on changing underlying assumptions and guided by an integrated knowledge translation (iKT) approach [19]. Core strategies included flexible facilitation and co-creation to support sustained change. The effectiveness of the interventions was associated with a reduced incidence of UC-UTIs from 18 to 4% [6], and in bladder distension from 41 to 9%, in patients with hip fracture [7], over four and five years, respectively.

This paper presents the process evaluation findings from the Safe Bladder intervention aiming to enhance understanding of barriers and enablers when implementing recommendations to prevent UC-UTIs and bladder distension. Specifically, we examined the implementation concepts of feasibility, acceptability and fidelity, guided by the following research questions:

1. To what extent was the intervention delivered as planned?
2. What factors influenced the implementation process, and how did these factors influence implementation outcomes?

## Methods

### Design

The study is a process evaluation of the *Safe Bladder* intervention, employing both a qualitative and a quantitative approach [20] and guided by the Medical Research Council (MRC) frameworks for process evaluation [8]. The evaluation focuses on the three core components of the intervention: (i) *Fidelity*,* dose and reach*; (ii) *Context;* and (iii) *Mechanisms of impact*. The effectiveness of the intervention, that is, the reduction in incidence of UC-UTIs and bladder distension, have been reported elsewhere [6, 7]. A logic model outlining the assumptions underpinning the intervention and its core components is presented in Additional file 1. Reporting this evaluation follows the Standards for Reporting Implementation Studies checklist [21], see Additional file 2.

### Setting and participants

The intervention was conducted at a university hospital in Sweden, performing ~ 10,000 orthopedic surgeries annually, including ⁓900 hip fractures. The participating units were selected based on their involvement in the care of patients aged ≥ 65 years with hip fractures: the emergency department (ED), operating room department (OR), post-anesthesia care unit/intensive care unit and three orthogeriatric wards. Approximately 400 registered nurses (RNs) and nurse assistants (NA) were employed across these units. In some units the RNs had special educations, e.g. nurse anesthetist and scrub nurses in the OR, critical care nurse in the post-anesthesia care unit/intensive care unit and surgical and trauma care in the ED.

Consistent with the iKT approach, each unit manager nominated two to three RNs and NAs with a particular interest in the area or informal leadership roles to participate in Learning Labs (Labs) as local facilitators, described below. In total 17 local facilitators were selected. The implementation program was led by four main facilitators who were researchers and/or clinicians at the study hospital. These included two specialist RNs with extensive experience in critical care and perioperative settings (MF, AEA), a consultant anesthetist (BN), and a gerontologist based in the orthogeriatric wards (AK).

### Tailoring of the intervention

The intervention was informed by classic theories on organizational culture and leadership, and dialogue [17], with a focus on changing underlying assumptions (Level 3) within the existing care culture, that is, how RNs and NAs think and act to prevent UC-UTIs and bladder distension. The main facilitators promoted safe, collaborative learning environments within the Labs and simulation-based training sessions. Dialogue techniques were employed to encourage shared reflection, active listening, and respect for diverse perspectives, thereby fostering collective learning and shared meaning-making [18]. This approach also aimed to enable shared meaning making, learning and collective thinking, with the participating stakeholders, described below. In keeping with the iKT approach, the intervention involved i partnership with shared decision-making, to increase relevance and uptake [19]. Flexible facilitation and co-creation were employed as core strategies to promote change and address barriers at macro (organizational), meso (departmental) and micro (individual) levels [22]. The education program included feedback on patient outcomes, case-based learning, goal setting, best practices recommendations, to prevent UC-UTIs and bladder distension, pre-optimization and fluid balance in.

### Intervention delivery

- Step (1) Adoption (2016–2017)** -** Introduction meetings, collaborations with stakeholders, that is, head of department; managers; front- and middle-line managers in involved units; manager for gerontologist, anesthesiologist and orthopedic surgeons; and quality and safety coordinators. Strategic decisions were taken, and goals were set.
- Step (2) Learning and adaptations (2017–2018)** -** Facilitation of Labs, meetings and education meetings in the units. Iteratively, co-creation of innovations: a *UC certificat*e and a *nurse-driven UC protocol.*
- Step (3) Implementation (2018–2019)** -** Implementing the UC certificate, five months, and testing the nurse-driven UC protocol and the bladder scanning schedule, 10 months.
- Step (4) Ownership (2019–2020)** -** Uptake of the innovations and if successful, ownership of the bladder bundle.

The bladder bundle was based on established best practices [1] and a modified version of the life cycle of a UC [23]. The modification consisted of a preceding step emphasizing a thorough patient assessment and patient involvement before selecting an appropriate UC method. In this study the context refers to internal or external factors, e.g. norms attitudes, routines, systems and resources which may facilitate or hinder implementation.

### Data collection

Process data were collected continuously from autumn 2016 to December 2019, by the main facilitators (MF) and (AE), depending on who was leading the learning activities. Qualitative data included field notes, implementation logbook written by the main facilitator (MF), memos, PowerPoint slides, email correspondence and observations form over 110 h of Labs, meetings and educations, simulation scenario training, lectures and clinical skill assessments. To assess dose and reach, quantitative data was collected including attendance rates of Labs, turnover rates, completion rates of UC-certificates, and adherence to the nurse-led UC protocol. These were documented and structured in an Excel file.

Aligned with the iKT and flexible facilitation approach, process data were regularly reviewed and discussed with main and local facilitators, as well as key stakeholders, to inform iterative adjustments in upcoming activities.

### Data analysis

Qualitative data were analyzed using deductive and inductive content analysis [20]. An analysis matrix, based on MRC guidance [8], was used to guide coding under the tree main categories: (i) Fidelity, dose and reach, (ii) Context, and (iii) Mechanisms of impact. The quantitative data [24], including attendance rates in the Labs, adherence to the innovations and staff turnover were tabulated under the predefined categories dose and reach [8]. The qualitative process data was compiled in a single document. The compiled data were read several times and sorted into content areas within the matrix by the first author (MF). The quotation was selected from the filed notes and memos and sorted within the matrix depending on its content. This process was confirmed by the last author (AE). Within the bound of the matrix the analysis proceeded, and different subcategories were created following the principles of inductive content analysis, first by the first author (MF), with rereading and discussion with the last author (AE). The subcategories were revised into the final subcategories after re-readings and discussions between the first and last authors. Final subcategories were agreed upon following consensus discussions with all authors to ensure trustworthiness. Three researchers (AE, EW and BG) had extensive experience in the field of implementation science and organizational change in complex environments and qualitative analysis, two (BN and CR) with extensive clinical research expertise, and one junior researcher (MF) was with clinical experience in critical and anesthesia care.

## Findings

The findings are presented in accordance with MRC guidance [8], organized into the three categories: (i), Fidelity, dose and reach, (ii) Context, and (iii) Mechanisms of impact, with six related subcategories.

### Fidelity, dose and reach

#### Engagement in learning labs and meetings

Overall, the intervention was delivered as planned, though it proved more time-consuming than anticipated. First, the iterative co-creation of the innovations was complex, partly due to the dual focus on preventing both UC-UTI and bladder distension. Second, modifying established catheter insertion techniques posed a greater challenge than expected. In response, and in keeping with the intervention’s flexible design, facilitators extended the planned five Labs to ten sessions before the innovations were implemented and tested. Attendance rates in the Labs and staff turnover are presented in Table 1. The reasons for not attending the Labs were mostly work-related, needed in clinical work.

**Table 1:** Attendance rate in the 10 learning labs, 17 months

Unit	Local facilitator	Attendance rate during the 10 Labs	Ending employment
Emergency room department	-Clinical instructor and registered nurse -Nurse assistant I^+^ -Nurse assistant (replaced nr I)	10 5 3	0 1 0
Ortho-geriatric ward I	-Registered nurse I -Registered nurse II	3 6	0 0
Ortho-geriatric ward II	-Registered nurse I -Registered nurse II^++^ -Registered nurse, replaced nurse II -Nurse assistant I (participating mid-intervention)	7 5 2 3	0 1 0 0
Ortho-geriatric ward III*	-Registered nurse I^+^ -Registered nurse II^+^ -Nurse assistant I^+^	3 4 2	1 (Not replaced) 1 (Not replaced) 1 (Not replaced)
Operating room department	-Operating room nurse I -Registered nurse anesthetist I	8 5	0 0
Post anesthesia care unit/intensive care unit	-Intensive care nurse (responsible for patient safety) -Nurse assistant I -Nurse assistant II	6 8 6	0 0 0
Sum	17	86	5

*Ward III merged with wards I and II

^++^Replaced due to other duties

^+^Ended employment during intervention

The main facilitators’ roles were to maintain a psychologically safe environment during Labs, free from shame or blame. Sessions opened with roundtable talk addressing implementation issues, followed by practical tasks such as reviewing the availability of sterile equipment and bladder scanners. Educational content included infection and bladder distension prevention, pre-optimization and fluid balance in elderly, regular feedback on patients’ outcomes (UC-UTIs and bladder distension), co-creation of the innovations and simulation scenario training. Between Labs, local facilitators undertook several tasks, including reflecting on UC practices, reinforcing aseptic insertion techniques, mentoring peers and leading simulation training and skill tests. Changes in practices before and after the intervention are presented in Table 2.

**Table 2:** Overview of changes to prevent UC-UTIs and bladder distension

Measures	Before intervention	After intervention
Changes to prevent UC-UTIs		
Staff training	Informal, word-of-mouth and proven skills. One of the wards used a theoretical test and a skill test	UC certificate (mandatory biennially), including learning material, a knowledge test, simulation scenario on low fidelity manikins, and a skill test
Equipment, sterility and preparation	Non-sterile	Aseptic
Pre-wash	Soap and water or sterile water or none	4% chlorhexidine soap sponge
Insertion techniques	Non-sterile gloves, non-sterile techniques	Sterile gloves, two persons, aseptic techniques
Changes to prevent bladder distension		
Catheter method	IC if retention, physician prescribe IUC, not using pre-defined IUC indications	A UC protocol with pre-defined indications to ensure the selection of an appropriate UC method.
Bladder scanning	6–8 h, act if ≥ 400 ml	Bladder scanning schedule adapted to fit the hospital’s bladder threshold: 100–150 ml: control after 3 h 150–300 ml: control after 2 h 300–400 ml: control after 1 h > 400 ml: IC or IUC, depending on patient assessment; see above
Catheter removal	Remove day 1 one post-surgery. A visual reminder.	Remove day one post-surgery. A visual reminder. Using predefined removal plans, and RNs can independently remove according to removal plan.
Patient involvement	Not standardized	Integrated into the UC-protocol

*Abbreviations*: *IC* intermittent catheterization, *IUC* indwelling urinary catheter, *RN* registered nurse, *UC* urinary catheterization

Education activities across units are detailed in Table 3, while communications and educational meetings with leaders, physicians and quality and safety coordinators, including turnover rates are presented in Additional file 3.

**Table 3:** Summary of educational meetings across units, April 2017 to May 2019

Units	Introductory ~ 60 min (MF, AEA, AK)	Infection Prevention, ~ 45 min (AEA, MF)	UC certificate ~ 60 min (MF)	Pre-optimization and fluid balance in elderly, ~ 45 min, (BN, MF)	UC-protocol, ~ 60 min (MF)	Preliminary outcomes, ~ 30 min (MF)
Emergency Department	3	2	2	2	2	1
Ortho-geriatric wards	6	1	8	2	4	1
Operating room department	3	Pre-intervention	2	Pre-intervention	2	2
Postanesthetic care unit/ICU	2	1	1	Pre-intervention	2	Feedback by local facilitators
Sum, *n* = 49	14	4	13	4	10	4
Component	Lecture and dialogue	Lecture and dialogue	Simulation and dialogue	Lecture and dialogue	Reviewing evidence, lecture Co-creation	Feedback and dialogue

*Main facilitators*: *MF* (ICU and nurse anesthetist), *AEA* (expert in infection prevention and change projects), *BN* (senior anesthesiologist) and AK (gerontologist)

*Abbreviations*: *ICU* intensive care unit, *UC* urinary catheter

We aimed for > 75% completion of the UC certificate (*n* = 401) within the five months of implementation but achieved 56% completion (range: 34–58%). However, the second innovation, i.e. implementation of the UC protocol, was more successful. Over the ten-month testing phase, all 586 patients were assessed correctly, and appropriate catheter methods were applied.

### Context

#### Leadership and staff turnover

Overall, the stakeholders supported the intervention and were active partners in the collaborative process. They acknowledge the range of the problems and appreciated the regular feedback on patient outcomes, not regularly reported before the intervention. However, we observed that they were not used to participating in interventions that promoted changes involving other clinical units. They also lacked a common platform for working with change projects. Moreover, turnover posed a major challenge: 32% (*n* = 43) stakeholders either changed roles or left their positions, see Additional file 3. Staff shortages led to the merging of two wards mid-intervention, increasing reliance on the main facilitators and hindering ownership of the intervention.

#### Time pressure, stress and peer support

Over time, local facilitators reported that the new practices were perceived as overly time-consuming and burdensome, competing with clinical demands. This contrasted with their initial enthusiasm and belief in the importance of reducing UC-UTIs and bladder distension. To address this barrier, the main facilitators introduced efficiency tips and ensured continued support in clinical skills. By the end of the intervention, this was not an issue of concern.

### Mechanism of impact

#### Factors that triggered change

Change was primarily driven by the combination of patient outcome feedback and patient case discussions, which made the link between past practices and adverse events more visible. Reflective discussions in Labs also encouraged changes in thinking. However, discussions often centered on peers’ mistakes (e.g. touching sterile catheters with contaminated gloves) rather than self-reflection. More open admissions of personal shortcomings occurred during small-group skills training:

RN 1: “The non-sterile technique is not safe!” I contaminate stuff all the time”.

#### Learning and relearning

Participants described the learning and unlearning process difficult and at times confusing. Previous shifts in catheterization practices had left staff unsure of the current best practices:

RN 2: “We learned the sterile way, then unsterile, and now it’s sterile again. Which is correct?”

RN 3: “One hospital uses 500 ml, another 300 ml for bladder threshold. We use 400 ml—what’s right?”

Facilitators supported this uncertainty by normalizing complexity and encouraging questioning. Moreover, the skill assessments were sometimes perceived as personal, i.e. not wanting to be assessed in UC clinical practices. The facilitators addressed this by sharing their own errors and fostering an environment of shared learning. Over time, confidence and acceptance of the new practices increased.

#### The importance of flexible facilitation and internal facilitators

The role of the main facilitators was critical throughout the multi-year intervention. Their responsiveness, close engagement with stakeholders, and consistent presence helped maintain momentum and address barriers. Local facilitators, in turn, contributed valuable contextual insights and were essential to co-creating the innovations and supporting sustainability. However, their ability to assume full ownership was affected by structural barriers. A key enabler of ongoing implementation was the decision by hospital managers and quality coordinators to continue the intervention beyond the study period.

## Discussion

To our knowledge, this is the first intervention underpinned by theories of organizational culture and leadership, and dialogue [17, 18] using an iKT approach [19], specifically targeting the prevention of both UC-UTI and bladder distension. Findings from this process evaluation suggest that the intervention was successful considering *feasibility*,* acceptability and fidelity*, factors that are crucial for long-term adoption and ownership of interventions [25]. Despite challenges such as staff turnover, the intervention reached over 50% of the participants with the UC certificate and all RNs followed the protocol during implementation. These fidelity outcomes, together with previously published clinical effects, i.e. reduction in UTI from 18 to 4% [6] and bladder distension from 41 to 9% [7], support the overall success of the intervention. Given the longitudinal design, the intervention allowed sufficient time for the new practices to be tested and adapted, which may support sustained changes in ‘the way we do things here’, a cultural element often transferred to new staff members [17]. The implementation strategies and theoretical framework employed enable the identification and overcoming of barriers across macro, meso and micro levels within the organization.

Consistent with earlier research [13, 26], key enablers included careful planning, early and continuous engagement with stakeholders, shared decision-making, goal setting, the involvement of local facilitators, and a flexible approach. The facilitators’ ability to create a psychologically safe space for learning enabled open dialogue, clarification of ambiguities, and collective meaning-making. These findings are in line with our previous experiences from the *Safe Hands* intervention [15, 16]. Moreover, flexible facilitation was essential for maintaining momentum throughout the year of implementation intervention. It also enabled us to mitigate contextual challenges including staffing shortages, time pressures, and high turnover, all of which are recognized as barriers when implementing complex interventions [12, 13, 27, 28]. These barriers reflect a broader reality across many healthcare systems, where resource constraints often obstruct the normalization of new routines and collective action [29]. Therefore, it is essential for healthcare organizations to provide adequate staffing and dedicated time for patient safety initiatives [29]. Further, despite its resource-intensive nature, our cost-effectiveness analysis showed that the intervention was less costly than standard care [30].

Other notable contextual challenges included a lack of familiarity with the iKT approach and absence of structured processes for collaborative change efforts. These organizational limitations delayed full ownership of the intervention. It is important to recognize that the structure, governance, and regulatory frameworks of healthcare systems affect resourcing, staff retention, and ultimately, the success of change initiatives [8, 25].

### The complexity of preventing urinary catheter associated infections

Urinary catheterization is a complex, multistep procedure, prone to errors of omission, and susceptible to variability in both practice and interpretation [31–35]. The UC model we used [23] addressed all critical steps for UC-UTI prevention. Our modified version included a preceding step that emphasized situational awareness and patient involvement, thereby supporting the appropriate catheter method selection [7]. To our knowledge, this model has been revised twice since the inception of our intervention, emphasizing a more strictly IUC avoidance approach [2, 14]. However, excessive avoidance of IUC can inadvertently lead to more frequent intermittent catheterizations and risk of bladder distension [6, 7]. Although intermittent catheterizations are often associated with lower infection risk and often excluded from prevalence and incidence metrics, our previous finding demonstrated 73% increased odds of developing a UTI per intermittent catheterization [6].

### Study strengths and limitations

A key strength of this study is the longitudinal nature of the intervention, during which we observed notable shifts in how RNs and NAs talked about and acted upon the prevention of UC-UTIs and bladder distension, consistent with the aims of the implementation program. We acknowledge that organizational policy changes may have impacted on our findings. While we cannot rule out the influence of broader organizational policy changes, our close collaboration with stakeholders suggests that any such changes would likely have been detected.

Due to the design of the study, it remains unclear whether we succeeded in altering HCPs underlying assumptions, the cultural taken-for-granted, as intended [17]. Nevertheless, the intervention has since been integrated into mandatory practice for all HCP at the study site, indicating likely behavioral and cultural shifts [36]. Furthermore, although we observed improvements in practice, we cannot confirm whether behaviors were changed consistently in daily routines. The potential influence of the Hawthorne effect, i.e. behavior change due to awareness of being observed, must also be considered [37] However, the substantial reduction in adverse events suggests that changes were sustained in routine care.

Several steps were taken to ensure trustworthiness of our findings. The process data were primarily collected by one of the main facilitators and researchers, rather than an external evaluator. While this approach enhances contextual understanding, it may introduce bias in both data collection and interpretation [8, 38]. However, in line with the iKT approach, process data were continuously discussed with stakeholders and local facilitators, guiding the ongoing intervention and enabling real-time identification of contextual barriers and facilitator [8, 25].

The MRC framework [8] was valuable for guiding the planning, execution, and analysis of process data. Nevertheless, in practice, it was sometimes difficult to clearly delineate between fidelity and mechanisms of impact. For example, high levels of fidelity, such as sustained participant engagement and adherence to intervention components, likely contributed to the observed outcomes, thereby serving as a mechanism of impact.

A limitation was the inability to conduct planned interviews with participants, which would have enriched the process evaluation and contributed to the credibility of the finding [38]. Additionally, we acknowledge that the findings may be specific to our context and participants, which limit the transferability to other settings.

## Conclusion

Implementing preventive measures for UC-UTIs and bladder distension in older patients with hip fractures is a complex and time-intensive endeavor. The application of theories on organizational culture and leadership, together with an iKT approach, was instrumental in facilitating the adoption of recommended preventive practices. These process findings can support healthcare leaders and decision-makers in planning and executing similar interventions in other clinical contexts.

Further research is needed to examine how staff turnover influences the sustainability of patient safety initiatives, particularly when reaching critical thresholds that may disrupt the routinization of new practices. Additionally, future studies could explore the generalizability of our approach to other patient populations, healthcare settings, and preventive interventions.

## Supplementary Information

Supplementary Material 1.

Supplementary Material 2.

Supplementary Material 3.

## Data Availability

“Data is provided within the manuscript and supplementary information files”.

## Abbreviations

HCP: Healthcare Professional
iKT: Integrated Knowledge Translation
IUC: Indwelling Urinary Catheter
MRC: Medical Research Council
NA: Nurse Assistant
OR: Operating Room Department
RN: Register Nurses
UC: Urinary Catheter
UTI: Urinary Tract Infections
