# Supplementary material for: Lesson learned from implementing measures to prevent urinary tract infection and bladder distension in patients with hip fractures - a process evaluation
Source: BMC Geriatr. 2025 Aug 2;25:584. doi: 10.1186/s12877-025-06216-w (PMC12318397; doi:10.1186/s12877-025-06216-w)
Supplement: Supplementary file 1 — Supplementary Material 1. [file 12877_2025_6216_MOESM1_ESM.docx]

| **Problem and evidence** | **Implementation intervention** | **Resources** | **Activities** | **Potential**  **mechanism of impact** | **Proposed outcomes** |
| --- | --- | --- | --- | --- | --- |
| **Problem**  -UC-UTI and bladder distension are common and largely preventable adverse events.  -Identified knowledge gap between preventive strategies and clinical practice  -Patients with hip fractures are vulnerable to adverse events.  -Unclear evidence which implementation theory and strategy is most effective in creating a sustained change in practices  **Evidence**  -Strategies to prevent UC-UTI and bladder distension include a bundle of measures | **Theories and strategies**  -Organizational culture and leadership, and dialogue. Creating a safe place for learning and thinking together  - Integrated Knowledge Translation  -Flexible facilitation  -Consider known barriers and enablers to change  - Goal settings  **Education**  -Infection prevention and UC-UTI and bladder distension prevention  -Refreshment of patient assessment tools, fluid balance in older patients and non-technical skills  **Implementation process**  -Emergent, agile and flexible facilitation | -Make videos on UC insertion  - External and internal facilitators and researcher with experience from implementation research and geriatric assessment, and one appointed doctoral student  -External fundings  -Adequate equipment, i.e. bladder scanner, catheterization kit, sterile gloves and simulation dummies  -Using local champions, i.e. expert nurses | -E-learning platform for UC certificate, i.e. theoretical test and skill test  -Learning Labs with expert nurses  -Education meetings  **Iteratively co-creating innovations**  1. UC certificate in the hospital’s e-learning platform, knowledge test and a skill test  2. A nurse-driven urinary catheter protocol and timely bladder scanning schedule to measure residual urine | -Creating a safe place for learning and thinking and co-creating together  -Agile, flexible and emergent approach.  -Integrated knowledge translation – context-specific knowledge that users contribute, important for sustainability  -Creating awareness of the problem and a sense of urgency  -The use of facilitation and facilitators  -Addressing barriers and enablers to change | -Change HCPs’ way of thinking and acting to prevent UC-UTI and bladder distension  -Increase HCPs’ knowledge in preventive strategies  - Reduced incidence of UC-UTI and bladder distension  -Improved nurse UC documentation  -Ownership of UC-UTI and bladder distension prevention  -Reduced costs |
| Process evaluation and intervention effectiveness in preventing UC-UTI and bladder distension and cost-effectiveness.  Data collection: Patient outcome from the local quality register, process data and interviews with the expert nurses. | | | | | |
| Abbreviations: HCPs = healthcare professionals, IUC = indwelling urinary catheter, UC = Urinary catheter, UTI = urinary tract infection, RN = Register nurses | | | | | |
| Supplementary file 2. Logic model for the Safe Bladder bundle intervention to increase adherence to measures to prevent UC-UTIs and bladder distension, version 7. | | | | | |
