# Supplementary material for: Lesson learned from implementing measures to prevent urinary tract infection and bladder distension in patients with hip fractures - a process evaluation
Source: BMC Geriatr. 2025 Aug 2;25:584. doi: 10.1186/s12877-025-06216-w (PMC12318397; doi:10.1186/s12877-025-06216-w)
Supplement: Supplementary file 3 — Supplementary Material 3. [file 12877_2025_6216_MOESM3_ESM.docx]

| **Additional file 3. Number of meetings and correspondence with stakeholders** | | | | | | |
| --- | --- | --- | --- | --- | --- | --- |
| **Stakeholders, N = 43** | **Autumn 2016** | **Winter/Spring 2017** | **Autumn**  **2017** | **Winter/Spring 2018** | **Autumn**  **2018** | **Spring**  **2019-2020** |
| Meeting content | Introduction, implementation plan, patient outcomes  ~60 minutes | Introduction, implementation plan and process, patient outcomes,  ~60 minutes | Implementation process and patient outcomes,  ~30 minutes | Implementation process and patient outcomes,  ~30 minutes | Implementation process and patient outcomes,  ~30 minutes | Preliminary process findings, ownership, patient outcomes,  ~30 minutes |
| Senior Managers n = 6 | | | | | | |
| OR, n = 1 | 1 |  | 1 |  |  | 1 + e-mail |
| Orthopaedic, n = 1 | 1 |  |  |  |  | e-mail |
| OGW, n = 1, retirement, new, n=1 | 1 |  | 1 |  | 1 (new) | 1 |
| ED, n = 1, ended employment, new, n = 1 | 1 |  | 1 (new) |  |  | 1 |
| Quality and safety coordinators, n=10 | | | | | | |
| Quality representatives, OGWs, n = 2, both ended employment, new, n = 2 | 1 | 1 | 1 (new) | 1 |  | e-mail |
| Quality and safety coordinators, n = 4, two ended employments new, n = 2 |  | 4 (separate meetings) |  | 1 (new) |  | e-mail |
| Frontline and middle-line managers, n = 27 | | | | | | |
| ED, n = 3 | 1 | 1 | e-mail | e-mail | e-mail | e-mail |
| OGW, n = 3, all ended employment, new, n = 3 | 1 | 1 (new) | e-mail | e-mail | e-mail | 1+e-mail |
| OR n = 4, all ended employment, new n = 4 | 1 | 1 (new) | 1+e-mail | 1+e-mail | 1+e-mail | 1+e-mail |
| PACU/ICU, n = 2, one ended employment, not replaced | 1 | 1 | e-mail | e-mail | e-mail | e-mail |
| Anaesthesiologist team leaders, n = 2, both ended employment, new, n=1 |  | 1 |  |  | 1 (new) | e-mail |
| Gerontologist team leader, n = 3 |  | 1 |  | 1 |  | e-mail |
| Trauma orthopaedic surgeon leader in ED, n =1 | 1 + e-mail | 1 + e-mail |  |  |  | e-mail |
| Orthopaedic surgeons responsible for hip fracture patients (also participated in Safe Hands), n = 1 | 1+ e-mail | 1+ e-mail |  |  |  | e-mail |
| Physicians team | | | | | | |
| The gerontologist team | 1 |  | 1 |  |  |  |
| The anaesthesiologist team |  | 1 |  | 1 |  | e-mail |
| The trauma orthopaedic surgeons’ team |  |  |  | 1 |  | e-mail |
| Total number of meetings (N = 46) | 12 | 14 | 6 | 6 | 3 | 5 |
| **Abbreviations:** ED =Emergency department, IUC = Intensive care unit, OR = Operating room department, OGW = Orthogeriatric wards, PACU = post-anaesthesia care units | | | | | | |
